# The Effect of Prognostic Communication on Patient Outcomes in Palliative Cancer Care: a Systematic Review

**DOI:** 10.1007/s11864-020-00742-y

**Published:** 2020-04-23

**Authors:** Naomi C. A. van der Velden, Maartje C. Meijers, Paul K. J. Han, Hanneke W. M. van Laarhoven, Ellen M. A. Smets, Inge Henselmans

**Affiliations:** 1 Department of Medical Psychology, Amsterdam University Medical Centers, Location Academic Medical Center, University of Amsterdam, Meibergdreef 9, 1105 AZ Amsterdam, The Netherlands; 2 Amsterdam Public Health Research Institute, Amsterdam, The Netherlands; 3 Cancer Center Amsterdam, Amsterdam, The Netherlands; 4 Center for Outcomes Research and Evaluation, Maine Medical Center Research Institute, Portland, OR USA; 5 Department of Medical Oncology, Amsterdam University Medical Centers, Location Academic Medical Center, University of Amsterdam, Amsterdam, The Netherlands

**Keywords:** Advanced cancer, Palliative care, Prognosis, Truth disclosure, Physician-patient communication, Patient outcomes

## Abstract

**Background:**

While prognostic information is considered important for treatment decision-making, physicians struggle to communicate prognosis to advanced cancer patients. This systematic review aimed to offer up-to-date, evidence-based guidance on prognostic communication in palliative oncology.

**Methods:**

PubMed and PsycInfo were searched until September 2019 for literature on the association between prognostic disclosure (strategies) and patient outcomes in palliative cancer care, and its moderators. Methodological quality was reported.

**Results:**

Eighteen studies were included. Concerning prognostic disclosure, results revealed a positive association with patients’ prognostic awareness. Findings showed no or positive associations between prognostic disclosure and the physician-patient relationship or the discussion of care preferences. Evidence for an association with the documentation of care preferences or physical outcomes was lacking. Findings on the emotional consequences of prognostic disclosure were multifaceted. Concerning disclosure strategies, affective communication seemingly reduced patients' physiological arousal and improved perceived physician’s support. Affective and explicit communication showed no or beneficial effects on patients’ psychological well-being and satisfaction. Communicating multiple survival scenarios improved prognostic understanding. Physicians displaying expertise, positivity and collaboration fostered hope. Evidence on demographic, clinical and personality factors moderating the effect of prognostic communication was weak.

**Conclusion:**

If preferred by patients, physicians could disclose prognosis using sensible strategies. The combination of explicit and affective communication, multiple survival scenarios and expert, positive, collaborative behaviour likely benefits most patients. Still, more evidence is needed, and tailoring communication to individual patients is warranted.

**Implications:**

Future research should examine the effect of prognostic communication on psychological well-being over time and treatment decision-making, and focus on individualising care.

## Introduction

Although antineoplastic treatment options have evolved in the past decades, cancer remains a leading cause of death globally [1–3]. To a certain degree, physicians are able to gain insight into advanced cancer patients’ prognosis and disclose the life-limiting nature of the disease. However, recent therapeutic developments and associated altered disease outcomes challenge oncologists’ prognostic assessments [4•]. Moreover, estimating an individual’s life expectancy is undeniably complex [4•, 5]. Nevertheless, in an era of patient autonomy and shared decision-making, physicians’ provision of prognostic information is considered important for patients to make informed treatment choices [6–9]. In addition, disclosure of prognosis might be necessary for them to prepare for the end of life [10]. A vast majority of patients with metastatic cancer wishes to be informed about the expected outcome of their illness [11, 12].

Yet, internationally, half of patients with advanced cancer are not aware of their prognosis [13–15]. Patients with incurable cancer often misunderstand the palliative intent of their treatment and overestimate their life expectancy compared with their oncologists [7, 13, 16–18]. What is more, some studies indicate that these misconceptions grossly remain unchanged over time as death approaches [19, 20].

A lack of prognostic awareness could lead to decisions in disaccord with patients’ actual values [17]. While accurate prognostic understanding is commonly associated with a preference for comfort care [17, 21], engagement in advance care planning [22, 23] and improved quality of life [24–28], misunderstanding of prognosis is associated with the administration of aggressive anticancer therapy [16, 17, 22, 29] and life-sustaining treatment [23, 25] at the end of life. Additionally, documentation of end-of-life preferences [22, 30] and usage of hospice services [30–33] can be complicated by prognostic unawareness.

Prognostic unawareness might result from the way physicians and patients communicate [34]. Oncologists are often reluctant to discuss prognosis [15, 34–37] and seem to worry about damaging the physician-patient relationship, patients’ hope or psychological well-being [6, 38, 39]. When oncologists provide prognostic information, current literature suggests that they use imprecise qualitative terms (e.g. “months to years”) instead of quantitative point estimates (e.g. means or medians) or survival rates (e.g. percentages) [34]. Furthermore, challenged with the fine art of balancing hope and honesty, physicians regularly emphasise the presence of beneficial prognostic markers, the best case scenario and “years” instead of “months” [34, 38, 40–42].

Most past research has focused either on the prevalence and consequences of prognostic unawareness or on patients’ information preferences, using observational or exploratory methods [43•]. However, information on how to best engage in prognostic discussions is limited. More specifically, few studies investigate the independent effect of prognostic communication on patient outcomes in palliative oncology, or factors that influence this relationship [34]. Hence, existing guidelines are mostly based on descriptive studies or expert consensus. Finally, there is no up-to-date synthesis of literature on this specific topic [44, 45].

Comprehensive information about the effect of different approaches to prognostic communication on advanced cancer patients is needed. This effort could assist oncologists in these challenging conversations, optimise the delivery of prognostic information and enhance patient outcomes. Integrating knowledge on moderating factors could help to tailor communication to individual patients, and ultimately formulate evidence-based advice for physicians’ clinical practice. Therefore, this systematic review addresses the following research questions:

I. What are the effects of prognostic disclosure on patient outcomes in palliative cancer care?
II. What are the effects of different strategies to prognostic disclosure on patient outcomes in palliative cancer care?
III. What patient and context characteristics moderate the effect of prognostic communication on patient outcomes in palliative cancer care?

## Methods

The Preferred Reporting Items for Systematic Reviews and Meta-Analyses (PRISMA) statement was used as a guideline for this systematic review [46]. No protocol was registered.

### Literature search

From July till September 2019, PubMed and PsycInfo databases were searched for studies published in English, using no restriction on publication year. The following search terms were used as main index terms or free-text words: “prognosis” and “communication” or “physician patient interaction” and “neoplasms” and “palliative care”. Additionally, synonyms and closely related words were used (Appendix 1). One recently published, not yet indexed article was found through a PubMed article alert.

### Eligibility criteria

Original quantitative studies describing the association between physician-patient communication about prognosis and patient outcomes in palliative cancer care were eligible. Palliative cancer care was defined as care for patients with incurable, metastatic cancer, including end-of-life care. Studies with samples partially matching the target population were only included if subgroup results were available. Communication about prognosis was defined as communicating the absence of cure, terminal nature of the disease and/or life expectancy. Papers addressing “bad news” without specifying its definition did not suffice. Communication could be real (e.g. self-reported by physicians or patients or observed in medical records, audio-recorded consultations or individually adapted consultations) or hypothetical (e.g. manipulated in video-recorded or written vignettes). Studies qualified if the independent effect of (strategies for) prognostic disclosure on (any type of) patient outcome(s) was examined. Hence, studies investigating more general interventions, such as advance care planning, early palliative care, decisions aids, question prompt lists or communication skills training, were excluded. Qualitative and non-empirical research, case reports and studies investigating minors or caregivers were also excluded.

### Study selection

Duplicates were removed. Two authors (NV, MM) double screened 10% of the resulting records based on title and abstract (*N* = 377). These authors independently agreed on the inclusion, exclusion or the necessity to retrieve full-text papers for 96% of this sample. For 4% (*N* = 15), judgement differed, but only on the necessity for further evaluation. The last author (IH) was involved to jointly decide on inclusion of papers with questionable eligibility and to specify the criteria. After resolving all differences, the remaining 90% (*N* = 3393) of records were screened individually based on title and abstract (NV, MM). Discussion between NV, MM and IH took place in case of doubt. These authors jointly evaluated potentially relevant papers in full text (*N* = 54) and decided on the final inclusion with 100% agreement.

### Data extraction

Data were extracted independently by NV and MM with a standardised extraction form including first author, year of publication, country, study aims, design, sample (description and size), setting, type of prognostic communication (prognostic disclosure and/or disclosure strategy), definition of prognosis (predictor), assessment of predictor, patient outcome and assessment and relevant main and moderating effects (direction and significance with *p* values or confidence intervals). Interaction terms and predictors of patients’ reactions to manipulated prognostic messages were regarded as moderating factors. The congruence of the independently extracted data was judged by MM. In case of doubt, discussion with NV, MM and IH took place until agreement was reached.

### Quality assessment

Appendix 2 displays the quality assessment tools used. The adapted Newcastle-Ottawa Scale was adjusted to assess the quality of non-experimental studies [47, 48]. Eight items were scored with 0 to 2 points, leading to a maximum score of 16. A quality checklist for experimental studies was self-developed to suit the various designs of the included studies. Items were based on the adapted Newcastle-Ottawa Scale [47, 48], the Cochrane Consumer and Communication Review Group criteria [49, 50] and the Cochrane Collaboration’s tool for assessing risk of bias [49]. Experiments using a within-subjects design were rated with 8 items (part A). Experiments using a between-subjects design were additionally assessed with 5 more items (part B). Items were scored with 0 or 1 point, leading to a maximum score of 8 or 13 points, respectively. Self-constructed items assessing the clarity of the definition of prognostic communication were included in both quality assessment tools.

Total scores and percentages of the maximum score were calculated. Papers attaining < 50% of the maximum score were considered of relatively low quality. Scores of ≥ 50% reflected satisfactory quality. Importantly, mutual comparison is only appropriate for studies using similar designs.

Methodological quality was assessed by NV, MM and IH. After these authors reached consensus during double assessment of one third of the papers (*N* = 6), MM and IH evaluated the remaining papers independently (*N* = 12). In case of doubt, discussion between all assessors took place. Agreement on quality was reached for all studies.

## Results

**Fig. 1:**
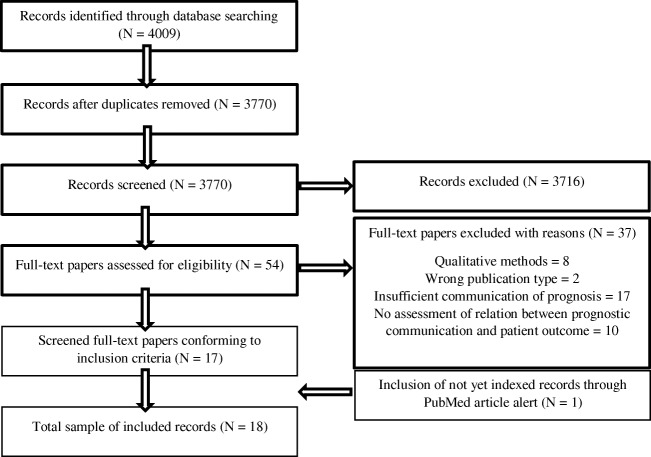
Flow diagram for article search and selection

The search yielded 3770 non-duplicate records (Fig. 1). Eighteen papers were included. Study characteristics are shown in Table 1.

**Table 1:** Characteristics and results of the included studies

Authors (year, country)	Study aims	Design	Sample	Setting	Type of prognostic communication	Definition of prognosis (predictor)	Assessment of predictor	Patient outcome and assessment	Relevant main effects^2^	Relevant moderating effects^3^
Y. Aoki et al. (1997, Japan)	To examine how disclosure of diagnosis, pathology and prognosis affects patient’s self-determination and attitude during the terminal stage	Cross-sectional study	12 patients with metastatic lung or breast cancer or sarcoma	1 academic medical centre	Prognostic disclosure	Prognosis^1^	Researcher-rated presence of prognostic disclosure (registered in medical records)	Length of last admission before death, sedation near death and choice of do-not-resuscitate order (registered in medical records)	No differences in length of the last admission before death, sedation near death or the choice of do-not-resuscitate order between prognostic disclosure vs. non-disclosure (*p* values NA) in bivariate analyses	NA
E.H. Bradley et al. (2001, USA)	To examine (1) the proportion of advanced cancer patients who have a documented prognostic discussion in their medical records, (2) the potential factors associated with such discussions, (3) the nature of such discussions and (4) the association between such discussions and advance care planning	Cross-sectional study	232 patients (≥ 65 years) with advanced brain, pancreas, liver, gall bladder or lung cancer	6 community hospitals	Prognostic disclosure	The word “prognosis”, time frames until death, life expectancy and expected disease outcome	Researcher-rated presence of prognostic discussion (registered in medical records)	Presence of do-not-resuscitate orders, discussions of life-sustaining treatment preference and advance directives^4^ (registered in medical records)	Positive associations between prognostic discussion and do-not-resuscitate orders (95% CI = 1.1 to 4.2) and discussions about life-sustaining treatments (95% CI = 2.8 to 12.0) No association between prognostic discussion and advance directives (95% CI = 0.4 to 1.4)	NA
L.D. Cripe et al. (2012, USA)	To examine (1) whether anxiety and depression relate to actual survival, patients’ or oncologists’ perceptions of prognosis or extent of prognostic discussions and (2) whether patient- or oncologist-reported extents of prognostic discussion moderate the association between patients’ perceptions of prognosis and anxiety or depression	Cross-sectional study	86 men with advanced cancer	1 cancer centre	Prognostic disclosure	Life expectancy	Patient- and oncologist-rated extent of prognostic discussion (self-constructed survey question)	Anxiety and depression (HADS); patient perception of life expectancy (survey question based on Weeks et al. [17, 18])	No association between patient-rated extent of prognostic discussion and anxiety (*p* = .318), nor between oncologist-rated extent of prognostic discussion and depression (*p* = .240) or anxiety (*p* = .967) in bivariate analyses Negative association between patient-rated extent of prognostic discussion and depression (none vs. full discussions, *p* = .028; brief vs. full discussions, *p* = .020) in multivariate analyses Interaction effect of patient-rated extent of prognostic discussion × patient-perceived prognosis on depression (more depression following a worse perceived prognosis with no or brief vs. full discussion, *p* = .011 vs. *p* = .188) Interaction effect of oncologist-rated extent of prognostic discussion × patient-perceived prognosis on anxiety (more anxiety following a worse perceived prognosis with full vs. no or brief discussion, *p* = .002 vs. *p* = .444) No interaction effect of oncologist-rated extent of prognostic discussion × patient-perceived prognosis on depression, or of patient-rated extent of prognostic discussion × patient-perceived prognosis on anxiety (*p* values NA)	NA
O.P. Danzi et al. (2018, Italy)	To examine (1) if the presence of supportive comments during a bad news consultation has a buffering effect on heart rate variability and (2) if it improves recall of provided information	Experiment (between-subjects design, RCT)	60 healthy women without previous cancer history	Experimental setting	Prognostic disclosure and disclosure strategy: affective vs. standard communication	Incurability, life expectancy and treatment options	Providing reassurance or not during a simulated bad news consultation (video-recorded vignettes^5^)	Heart rate variability categorised in frequency and time parameters^6^ (ECG signals from the ECG100C Electrocardiogram Amplifier); doctor empathy, doctor support, doctor engagement, personal involvement, self-perceived recall ability (self-constructed survey questions); recall (survey questions based on Sep et al. [53])	High-frequency power decreased (*p* < .005), and low-frequency/high-frequency ratio increased (*p* < .05) only in the standard condition in bivariate analyses, during disclosure of incurability before the manipulation started After the manipulation, during communication about life expectancy and treatment options, there were higher rates of high-frequency power (*p* = .041) and low-frequency power (*p* = .027) in the standard vs. affective condition in bivariate analyses, as well as more perceived doctor empathy (*p* = .037) and doctor support (*p* < .001) in the affective vs. standard condition No differences in doctor engagement (*p* = .051), personal involvement, self-perceived recall ability or recall (*p* values NA) between the affective vs. standard condition after the manipulation in bivariate analyses Interaction effect of the manipulation × heart rate variability on recall (better recall of central prognostic information and additional treatment information following increased standard deviation of the inter-beat interval with standard vs. affective communication, *p* = .002 and *p* = .030; better recall of additional treatment information following increased high-frequency power with affective vs. standard communication, *p* = .047)	NA
A.C. Enzinger et al. (2015, USA)	To examine (1) the proportion of patients who want to know their life expectancy and who report that their physician disclosed a prognostic estimate and (2) whether prognostic disclosure is associated with more accurate patient perceptions of life expectancy and more frequent advance care planning without harm to patients’ well-being or the patient-physician relationship	Cohort study	590 patients with metastatic solid malignancies with progressive disease after ≥ 1 line of palliative chemotherapy	6 cancer centres (outpatient clinic)	Prognostic disclosure	Life expectancy	Patient-rated presence of prognostic disclosure (self-constructed interview question)	Survival (registered in medical records); prognostic understanding of life expectancy and health status (self-constructed interview questions); sad/depressed mood and worried/anxious mood (MQOL psychological subscale); major depressive disorder and generalised anxiety disorder (SCID-5 and Endicott scale); patient-physician relationship (self-constructed interview questions)	No difference in survival between prognostic disclosure vs. non-disclosure (*p* = .07) in bivariate analyses Positive association between prognostic disclosure and accuracy of life expectancy self-estimates within 3 months, 6 months and 12 months of actual survival (95% CI = 0.78 to 4.12; 95% CI = 1.07 to 3.73; 95% CI = 1.11 to 3.51) in bivariate analyses, particularly preventing gross overestimation of > 2 years and > 5 years (95% CI = 0.14 to 0.82; 95% CI = 0.08 to 0.47) Negative association between prognostic disclosure and length of life expectancy self-estimates (*p* = .0024) and positive association between prognostic disclosure and terminal illness acknowledgement (95% CI = 2.00 to 5.15) in multivariate analyses No associations between prognostic disclosure and sadness/depressed mood (*p* = .299), worried/anxious mood (*p* = .255), major depressive disorder (95% CI = 0.83 to 4.14), generalised anxiety disorder (95% CI = 0.04 to 2.44) or patient-physician relationship (95% CI = 0.75 to 1.93) in multivariate analyses	NA
A.S. Epstein et al. (2016, USA)	To examine the effects of recent and past clinical prognostic discussions on changes in illness understanding by advanced cancer patients	Cohort study	178 patients with advanced cancer refractory to prior chemotherapy whom oncologists expected to die within 6 months	9 cancer centres	Prognostic disclosure	Prognosis^1^ and life expectancy	Patient-rated presence of discussion of prognosis or life expectancy during the last or past visits (self-constructed interview questions)	Changes in illness understanding from pre- to post-scan visit (self-constructed interview questions)	Positive changes in illness understanding in groups reporting recent (*p* = .002) and recent and past (*p* = .028) discussions of prognosis or life expectancy No changes in illness understanding in groups reporting only past (*p* = .221) or no (*p* = .885) discussions of prognosis or life expectancy	NA
J.J. Fenton et al. (2018, USA)	To examine whether 2 measures of prognostic discussion were associated with deleterious pre- to post-visit changes in advanced cancer patients’ ratings of their relationship with their oncologists	Cohort study (baseline of RCT)	265 patients with stage III or IV non-haematological cancer whose oncologists would not be surprised if the patient died within 12 months	4 community-based cancer clinics, 3 community hospitals and 3 academic medical centres	Prognostic disclosure	Incurability, life expectancy, likelihood of effective treatment and transition from active to palliative treatment	Patient-rated presence of discussion of life expectancy (self-constructed interview question) and coding of prognostic discussions during audio-recorded visits (adapted PTCC informing subscale)	Changes in perceived strength of patient-oncologist relationship from baseline to 2 days to 7 days and to 3 months after patients’ visit, categorised in therapeutic alliance (THC) and confidence in obtaining information and attention of physicians (PEPPI)	No associations between coded prognostic discussion and changes in perceived patient-oncologist relationship (THC, *p* = .09; PEPPI, *p* = .84) from 2 days to 7 days Positive association between coded prognostic discussion and changes in perceived therapeutic alliance (*p* = .029) from baseline to 3 months No associations between coded prognostic discussion and changes in perceived confidence in obtaining information and attention of physicians (*p* = .73), nor between patient-reported prognostic discussion and changes in perceived strength of patient-oncologist relationship (THC, *p* = .21; PEPPI, *p* = .13) from baseline to 3 months	NA
K. Fletcher et al. (2013, USA)	To examine (1) gender differences in advanced cancer patients’ understanding of their illness and (2) gender differences in patients’ reports of discussions of life expectancy with their oncologists and (3) its effect on differences in illness understanding	Cohort study	68 patients with advanced cancer refractory to prior chemotherapy whom oncologists expected to die within 6 months	5 comprehensive cancer centres	Prognostic disclosure	Prognosis^1^ and life expectancy	Patient-rated presence of discussion of prognosis or life expectancy during the last or past visits (self-constructed interview questions)	Changes in illness understanding from pre- to post-scan visit, categorised in the acknowledgement of terminal disease, incurable disease and disease stage (self-constructed interview questions)	Positive associations between discussions of life expectancy or prognosis and terminal illness acknowledgement (*p* = .036) and a trend for recognition of incurable disease (*p* = .085) controlled for gender differences No associations between discussions of life expectancy or prognosis and knowledge of advanced disease stage (*p* = .506) controlled for gender differences	NA
R.G. Hagerty et al. (2005, Australia)	To examine (1) the context and way in which incurable metastatic cancer patients want to be informed about prognosis and (2) what features in the delivery of prognostic information they would experience as more or less hopeful	Experiment (within-subjects design)	126 patients with consecutive metastatic cancer who were diagnosed within 6 weeks to 6 months before recruitment	12 medical centres (outpatient clinic)	Disclosure strategy: conveying vs. discouraging hope	Prognosis^1^	Providing communication behaviours of physicians that might convey or discourage hope (written vignettes)	Hopefulness (survey questions based on Butow et al. [52] and Sardell and Trierweiler [51])	Rated as most hopeful communication behaviours were offering the most up-to-date treatment (90%), appearing to know all there is to know about the patient’s cancer (87%), occasional use of humour (80%), telling that the pain will be controlled (87%) and telling all treatment options (83%), shown in univariate analyses Rated as not hopeful were appearing to be nervous or uncomfortable (91%), giving prognosis to family first (87%), use of euphemisms (82%), avoiding talking about cancer and only discussing treatment (75%) and giving good news first and then bad news (72%) Giving statistics about life expectancy was rated evenly hopeful (30%), not hopeful (32%) and neutral (38%) in univariate analyses, just like expressing uncertainty about the disease course (35% vs. 30% vs. 35%)	Age was a predictor of rating the expert/positive/collaborative and empathic approach as hopeful (more hope following expert/positive/collaborative and empathic approach among older vs. younger patients, *p* = .04 and *p* = .002) Anxiety was a predictor of rating the expert/positive/collaborative and empathic approach as hopeful (more hope following expert/positive/collaborative and empathic approach among more vs. less anxious patients, *p* = .03 and *p* = .02) No associations between age or anxiety and rating the avoidant approach as hopeful, nor between sex, relationship status, religiosity, language, expected survival, time since metastatic diagnosis or involvement preferences and rating the expert/positive/collaborative, empathic or avoidant approach as hopeful (*p* values reported)
B.E. Kiely et al. (2013, Australia)	To examine the attitudes of people with a cancer experience to using 3 scenarios for survival to present information about life expectancy to patients with advanced cancer	Experiment (within-subjects design)	505 oncology clinic attendees (251), diagnosed with all types and stages of cancer, and women with a history of breast cancer (254)	2 general hospitals (outpatient clinic) and 1 consumer group	Disclosure strategy: worst, typical and best case scenario vs. median survival	Life expectancy	Providing the shortest 5–10%, middle 50% and longest 5–10% of survival time or estimated median survival time (written vignettes)	Attitudes to different types of prognostic information categorised in making sense, being helpful, being helpful to make plans for the future, helping family members and carers, conveying hope, taking hope away, being reassuring, being upsetting, decreasing anxiety, increasing anxiety, improving of understanding of survival time and preference to be included when explaining life expectancy (self-constructed survey questions)	More patients agreed that explaining 3 scenarios vs. median survival would make sense (93% vs. 76%), be helpful (93% vs. 69%), help making plans for the future (88% vs. 70%), help family and carers (91% vs. 71%), convey hope (68% vs. 44%), be reassuring (60% vs. 40%), decrease anxiety (43% vs. 32%) and improve understanding of survival time (93% vs. 75%) (all *p* < .001) in bivariate analyses Fewer patients agreed that explaining 3 scenarios vs. median survival would take away hope (11% vs. 24%), be upsetting (24% vs. 36%) and increase anxiety (22% vs. 34%) (all *p* < .001) in bivariate analyses	Education was a predictor of agreeing that the best case scenario conveyed hope (more agreement among higher vs. lower educated patients, *p* = .006) and should be included (more agreement among higher vs. lower educated patients, *p* = .001) in bivariate analyses Education was a predictor of agreeing that the typical scenario should be included (more agreement among higher vs. lower educated patients, *p* = .05) and 3 scenarios were helpful (more agreement among higher vs. lower educated patients, *p* = .05), but not of agreeing that 3 scenarios made sense/were easy to understand (*p* = .50) in bivariate analyses Cancer type was a predictor of agreeing that 3 scenarios were reassuring (less agreement among breast cancer vs. other types, *p* = .009) and upsetting (more agreement among breast cancer vs. other types, *p* < .001) in multivariate analyses Gender was a predictor of agreeing that 3 scenarios were helpful to make plans for the future (more agreement among females vs. males, *p* = .009) and worst case scenario was upsetting (more agreement among females vs. males, *p* = .02) in multivariate analyses Time since cancer diagnosis and age were predictors of agreeing that worst case scenario was upsetting (more agreement among patients diagnosed > 1 year vs. ≤ 1 year ago, *p* = .006; more agreement among patients ≤ 50 vs. ≥ 70 years, *p* = .001; more agreement among patients 51–69 vs. ≥ 70 years, *p* = .06) in multivariate analyses No associations between respondent characteristics and agreeing that all 3 scenarios conveyed hope, made sense or increased anxiety (*p* values NA)
M. Mori et al. (2019, Japan)	To examine (1) the effect of explicit prognostic disclosure on uncertainty at the time of cancer recurrence, (2) whether explicit prognostic disclosure improves patient satisfaction without worsening anxiety and (3) whether it improves patient self-efficacy	Experiment (within-subjects design)	105 women with breast cancer who had undergone curative surgery in a comprehensive cancer centre	Experimental setting	Disclosure strategy: more vs. less explicitness	Life expectancy	Providing numbers or not (e.g. 2-year survival rate) during a simulated bad news consultation (video-recorded vignettes^5^)	Uncertainty and self-efficacy (survey questions based on Van Vliet et al. [54, 55]); satisfaction (PSQ); anxiety (STAI-state); willingness to discuss advance care planning (self-constructed survey question)	Less uncertainty (*p* = .032) and more satisfaction (*p* = .010) with more vs. less explicitness in bivariate analyses No differences in anxiety (*p* = .198), self-efficacy (*p* = .277) or willingness to discuss advance care planning (*p* = .240) with more vs. less explicitness in bivariate analyses No associations between explicitness and uncertainty (*p* = .074), satisfaction (*p* = .78), anxiety (*p* = .461), self-efficacy (*p* = .432) or willingness to discuss advance care planning (*p* = .371) in multivariate analyses	NA
N. Nakajima et al. (2012, Japan)	To examine the association between specific information provided for patients with cancer and the quality of terminal care in patients and their families	Cross-sectional study	87 patients with terminal cancer who died during the last 27-month period	1 general hospital	Prognostic disclosure and disclosure strategy: more vs. less specificity	Incurability and life expectancy	Researcher-rated level of prognostic communication categorised in (A) non-disclosure of cancer diagnosis; (B) disclosure of cancer diagnosis; (C) disclosure of life-threatening diagnosis, e.g. metastasis and incurability; and (D) additional disclosure of poor prognosis, e.g. life expectancy (registered in medical records)	Health care provider perception of quality of terminal care categorised in psychological state, recognition of disease condition, communication and physical symptoms (STAS-J)	Less physical, behavioural and concentration-related symptoms of anxiety in patients (*p* = .0201) and family (*p* = .0240) with disclosure vs. non-disclosure of incurability (B vs. C) in bivariate analyses Better recognition of disease condition in patients (*p* < .0001), as well as better communication among medical staff, between patient and family and between patient, family and medical staff (all *p* < .0001), with disclosure vs. non-disclosure of incurability (B vs. C) in bivariate analyses Better recognition of disease condition in patients (*p* < .0001) and family (*p* < .0001), as well as better communication among medical staff, between patient and family, and between patient, family and medical staff (all *p* < .0001), with more vs. less specificity (C vs. D) in bivariate analyses No differences in physical symptoms, or pain or other symptoms specifically, in patients (*p* = .1860) or family (*p* = .1892) between levels of prognostic communication in bivariate analyses	NA
T.M. Robinson et al. (2008, USA)	To examine (1) patient-oncologist pairs with concordant and disconcordant views of prognosis and (2) the communication factors that may influence concordance about chance of cure	Cross-sectional study (baseline of RCT)	141 patients with advanced cancer whose oncologist would not be surprised if the patient was admitted to the intensive care unit or died within 12 months	2 academic medical centres and 1 veterans hospital	Disclosure strategy: optimistic vs. pessimistic statements about the past, present or future vs. uncertain statements	Incurability, disease course and disease outcome	Coding of optimistic, pessimistic or uncertain statements about the past, present or future during audio-recorded discussions of test results, treatment or prognosis (frequency counts)	Physician-patient concordance about chance of cure based on physician and patient perception of cure 10 days post-visit (self-constructed survey question)	No associations between statements of optimism in total (*p* = .198) and about the past/present (*p* = .700) and future (*p* = .704), pessimism about the past/present (*p* = .198) or uncertainty (*p* = .988) and patient-physician concordance about chance of cure in bivariate analyses Positive association between statements of pessimism in total (*p* = .006) and about the future (*p* = .017) and patient-physician concordance about chance of cure in multivariate analyses	NA
T. Rumpold et al. (2015, Austria)	To examine (1) the information preference of advanced lung cancer patients regarding cure rates and prognosis, (2) patients’ satisfaction with an individually adapted medical consultation and (3) patients’ emotional responses to the information	Quasi-experiment (allocation based on patient preference)	50 patients with advanced lung cancer	1 academic medical centre	Prognostic disclosure and disclosure strategy: qualitative vs. qualitative and quantitative information	Incurability and life expectancy	Providing cure rates, cure rates and life expectancy, or none, and providing additional quantitative as well as qualitative information (e.g. 5-year survival rate or median survival time) or not (individually adapted medical consultation)	Satisfaction and emotional response categorised in relief/distress, clarity/confusion, reduced/increased anxiety, security/insecurity, strengthened/weakened confidence and feeling supported/overwhelmed (self-constructed survey questions)	More distress in patients with requested disclosure vs. non-disclosure of cure rates and/or life expectancy (*p* = .009), but no differences in confusion, anxiety, insecurity, confidence, feeling overwhelmed or satisfaction (*p* values NA) in bivariate analyses No differences in emotional response or satisfaction (*p* values NA) between requested qualitative vs. qualitative and quantitative information in bivariate analyses	NA
M.S.C. Sep et al. (2014, The Netherlands)	To examine (1) whether clinicians can lower patients’ physiological arousal and (2) whether they can improve recall of provided information in a bad news consultation by means of affective communication	Experiment (between-subjects design, RCT)	50 healthy women without previous cancer history	Experimental setting	Prognostic disclosure and disclosure strategy: affective vs. standard communication	Incurability, life expectancy and treatment options	Providing reassurance or not during a simulated bad news consultation (video-recorded vignettes^5^)	Skin conductance level (microsiemens from the BIOPAC MP150); recall (self-constructed survey questions); non-abandonment, reassurance of support and doctor empathy (adapted QUOTE-COM)	Skin conductance level increased (*p* = .004) in the affective and standard condition in bivariate analyses, during disclosure of incurability before the manipulation started After the manipulation, during communication about life expectancy and treatment options, there was better recall of information (*p* = .035), more perceived doctor non-abandonment (*p* = .002) and reassurance (*p* = .02), as well as a trend for doctor empathy (*p* = .08), in the affective vs. standard condition in bivariate analyses Stronger decrease of skin conductance level (*p* < .0001) in the affective vs. standard condition, as well as an association between skin conductance level and recall (*p* = .01) only in the affective condition, after the manipulation in multivariate analyses	NA
J.A. Shin et al. (2016, USA)	To examine (1) quality of life, depression, anxiety and perceptions of prognosis in patients with metastatic breast cancer and (2) whether symptom burden and prognostic understanding differed between patients receiving endocrine therapy and chemotherapy	Cross-sectional study	140 patients with metastatic breast cancer receiving either endocrine therapy (40) or chemotherapy (100)	1 cancer centre (outpatient clinic)	Prognostic disclosure	Prognosis^1^	Patient-rated frequency of prognostic conversations (PTPQ single item)	Anxiety and depression (HADS)	Negative association between frequency of prognostic conversations and depressive symptoms (*p* < .01) No association between frequency of prognostic conversations and anxiety (*p* value NA)	NA
L.M. Van Vliet et al. (2013, The Netherlands)	To examine the effect of more vs. less explicit prognostic information and reassurance about non-abandonment at the transition to palliative care	Experiment (within-subjects design)	104 patients with or survivors of breast cancer (51) and healthy women (53)	Experimental setting	Disclosure strategy: more vs. less explicitness and affective vs. standard communication	Life expectancy and treatment options	Providing numbers or not (e.g. 2-year survival rate) and providing reassurance or not during a simulated bad news consultation (video-recorded vignettes^5^)	Uncertainty and self-efficacy (self-constructed survey questions); anxiety (STAI-state); satisfaction (PSQ)	Negative associations between explicitness and uncertainty (*p* < .001), but not anxiety (*p* = .562) Positive associations between explicitness and self-efficacy (*p* = .004) and satisfaction (*p* < .001) Negative associations between affective communication and uncertainty (*p* = .002) and anxiety (*p* = .001) Positive associations between affective communication and self-efficacy (*p* < .001) and satisfaction (*p* ≤ .001) Lowest uncertainty and anxiety and highest self-efficacy and satisfaction for high explicitness and affective communication (all outcomes *p* ≤ .05, except anxiety, *p* = .06)	Monitoring coping style was a moderator of the association between explicitness and uncertainty, anxiety, self-efficacy and satisfaction (more uncertainty and anxiety and less self-efficacy and satisfaction following explicitness among high monitors vs. low monitors, *p* = .007, *p* = .007, *p* = .012 and *p* = .048) No moderating effect of blunting coping style on the association between explicitness and patient outcomes, nor an effect of monitoring or blunting coping style on the association between affective communication and patient outcomes (*p* values reported)
G.J. Wagner et al. (2010, USA)	To examine how provider communication and patient understanding of life-limiting illness relates to patient discussion of care preferences with providers and family by studying how often these elements of communication take place and studying the associations among them	Cross-sectional study (baseline of RCT)	400 inpatient veterans with a life-limiting illness (260 having cancer, 224 having a non-cancerous disease)	1 veterans hospital	Prognostic disclosure	Life-limiting nature of the disease	Patient-rated presence of prognostic discussion (interview questions based on Quirt et al. [9])	Prognostic understanding (interview question based on Quirt et al. [9]); discussion of care preferences with provider and family and documentation in a living will (self-constructed interview questions)	Better prognostic understanding in patients with vs. without prognostic discussion (*p* < .001) among the total sample in bivariate analyses and among non-cancer patients and cancer patients separately (*p* values NA) in subgroup analyses More discussion of care preferences with family (*p* < .01), as well as a trend for these discussions with providers (*p* < .10), in patients with vs. without prognostic discussion among the total sample in bivariate analyses No association between prognostic discussion and discussion of care preferences with family (*p* = .21) or providers (*p* = .73), or documentation of care preferences in a living will (*p* = .18) among the total sample in multivariate analyses	NA

*CI* confidence interval, *ECG* electrocardiogram, *HADS* Hospital Anxiety and Depression Scale, *MQOL* McGill Quality of Life Questionnaire, *NA* not available, *PEPPI* Perceived Efficacy in Patient-Physician Interactions scale, *PSQ* Patient Satisfaction Questionnaire, *PTCC* Prognostic and Treatment Choices scale, *PTPQ* Prognosis Treatment and Perceptions Questionnaire, *QUOTE-COM* Quality of Care Through the Patient’s Eyes, *RCT* randomised controlled trial, *SCID-5* Structured Clinical Interview for DSM-5, *STAI-state* State-Trait Anxiety Inventory state version, *STAS-J* Support Team Assessment Schedule–Japanese, *THC* The Human Connection scale, *vs.* versus

^1^Prognosis as predictor was not further defined

^2^Results of multivariate analyses were reported unless otherwise specified

^3^Moderating effects encompassed interaction effects and predictors of patients' reactions to manipulated prognostic messages

^4^Advance directives encompassed a living will, health care proxies and durable power of attorney for health care forms [56]

^5^Video-recorded vignettes used comparable scripts based on one previous qualitative study [55], all including disclosure of incurability before manipulation with explicit (life expectancy) and/or affective (life expectancy and/or treatment options) communication. Van Vliet et al. [54] and Mori et al. [57] examined the effect of the manipulation only. Sep et al. [53] and Danzi et al. [58] reported on of the effect of disclosing incurability additionally

^6^Time domain parameters from heart rate variability series were the mean value of inter-beat intervals and the standard deviation. The median value along time was considered for further analysis. Frequency domain parameters from heart rate variability series were the median power spectral density of 5-s moving windows within two bandwidths: low-frequency (from 0.04 to 0.15 Hz) and high-frequency (from 0.15 to 0.4 Hz) bandwidths. low-frequency reflects sympathetic activity with some degree of parasympathetic measure, and high-frequency derives from vagal or parasympathetic activity. The ratio between low-frequency and high-frequency is regarded as an index of sympathetic-parasympathetic balance on heart rate modulation and reflects the sympathovagal interaction of the autonomic nervous system [58]

### Quality assessment

Considering the twelve non-experimental studies, the definition of prognosis, sampling strategy, use of validated tools for patient outcomes, statistical tests and controlling for confounders were often satisfactory. However, a justified and satisfactory sample size, and comparability of responders and non-responders, or of responders with the target population, was frequently lacking. Hence, there might be problems with power and selection bias. Eight non-experimental papers attained ≥ 50% of the maximum score. Four studies had relatively low quality (Table 2 in Appendix 3).

The manipulation of the predictor was well described in all six experimental studies; the definition of prognosis was clear in most. Additionally, randomisation, allocation, blinding of patients, comparison of groups and presence of equivalent conditions were adequate in both of the controlled trials. Blinding of the data analyst was not done in any of the experiments, and most lacked comparison of responders and non-responders, or comparison with the target population. Altogether, six papers showed methodological quality of ≥ 50% (Table 3 in Appendix 4).

### Prognostic disclosure

The effect of disclosing prognosis on advanced cancer patients was investigated by thirteen studies. Patient outcomes were categorised into information-related outcomes (e.g. understanding of prognosis), physical outcomes (e.g. symptoms), physiological outcomes (e.g. arousal), psychological outcomes (e.g. depression), relational outcomes (e.g. physician-patient relationship) or care preferences (e.g. documentation of treatment choices).

#### Information-related outcomes

Five studies assessed the association between disclosure of incurability and/or life expectancy and patients’ prognostic understanding. All papers demonstrated significant positive associations [22, 59–62], although two examined a partially similar sample [59, 62]. Furthermore, a significant negative association between disclosure of life expectancy and length of patients’ life expectancy self-estimates, and a positive association with the accuracy of these estimates, was found [22].

#### Physical outcomes

One study investigated the association between disclosure of incurability and physical symptoms, such as pain, and did not show a significant difference [60].

#### Physiological outcomes

Two studies examined the association between disclosure of incurability and physiological outcomes. In the experiment of Sep et al. [53], a significant increase of skin conductance was detected after physicians revealed the absence of cure in a simulated bad news consultation. In a similar experiment, Danzi et al. [58] studied activation of the autonomic nervous system through cardiac measures. Findings suggested significantly reduced parasympathetic activity, as well as increased sympathetic activity, among a part of the sample following disclosure of incurability [58]. Increased physiological arousal, revealed in both studies, can be regarded as a marker of emotional stress [53, 58].

#### Psychological outcomes

Five studies investigated the association between prognostic disclosure and psychological outcomes, of which two had relatively low quality (< 50%) [63, 64]. Cripe et al. [63] discovered a significant negative association of patient-rated extent of discussions about life expectancy with depressive symptoms. Moreover, depressive symptoms were less prevalent among respondents perceiving a worse prognosis when a full (versus no or a brief) discussion about life expectancy had taken place [63]. Similarly, more conversations about the likely disease outcome were significantly associated with less depressive symptoms [65]. Regarding anxiety, significantly lower levels were found in patients and their family when incurability was disclosed [60]. Other findings did not show differences in depression [22, 63], anxiety [22, 63–65] or feelings of confusion, insecurity, support or confidence in relation to prognostic discussions [64]. In contrast with previous findings, Cripe et al. [63] additionally revealed significantly more anxiety among respondents who perceived a worse prognosis after a full (versus no or a brief) discussion about life expectancy. Another study discovered more distress in patients who requested cure rates and survival estimates during an individually adapted consultation compared with patients who did not [64].

#### Relational outcomes

Four studies examined the association of prognostic conversations with relational outcomes. One showed a significant positive association between the disclosure of incurability and communication between patients, family and professionals [60]. Another study demonstrated significantly increased therapeutic alliance 3 months after discussions about incurability and survival [66]. Therapeutic alliance did not change from baseline to 2 days to 7 days after the consultation, nor did patients’ confidence in obtaining information and attention of their physicians at any time point [66]. A third paper revealed no significant association between conversations about life expectancy and the physician-patient relationship [22]. Finally, satisfaction with an individually adapted consultation did not differ between patients receiving requested information about cure rates and life expectancy and patients who denied such information [64].

#### Care preferences

Three studies assessed the association between prognostic disclosure and care preferences. Bradley et al. [56] demonstrated that patients reporting conversations about life expectancy were more likely to have had physician-patient discussions about life-sustaining treatment preferences. In a sample mainly consisting of terminal cancer patients, a significant association between the disclosure of patients’ life-limiting illness and discussing care preferences with family, as well as a positive trend for this discussion with physicians, was observed in bivariate analyses only [61]. One study additionally revealed a significant association between disclosure and the documentation of a do-not-resuscitate order [56]. Another paper, with relatively low quality, could not confirm this result, nor were significant differences found in length of the last admission before death or the administration of sedation near death [67]. No significant associations between prognostic disclosure and documentation of a living will, health care proxies or durable power of attorney for health care were reported either [56, 61].

### Disclosure strategies

Different strategies for prognostic communication, potentially influencing the effect of disclosure, were investigated by nine studies. Disclosure strategies were categorised into the provision of explicit prognostic information, framing of prognostic information, affective communication and general communication behaviours. Patient outcomes were categorised as previously reported.

#### Explicitness of prognostic information

Five papers assessed the association between explicit prognostic information and patient outcomes. Explicit communication encompassed disclosure of more specific prognostic information (e.g. life expectancy in addition to incurability) [60] or provision of quantitative (e.g. survival rate, median survival time and/or range) instead of, or supplemental to, qualitative survival information [54, 57, 64, 68].

Considering *information-related outcomes*, one cross-sectional study noted significantly better recognition of disease condition among patients who received more specific prognostic information [60]. Concerning *physical outcomes*, specific prognostic information was not associated with physical symptoms in general, nor with pain or any other specific symptoms [60]. Regarding *psychological outcomes*, uncertainty was significantly lower after the communication of quantitative versus qualitative information (in bivariate [57] and multivariate [54] analyses). Van Vliet and colleagues [54] additionally revealed enhanced self-efficacy regarding patients’ ability to deal with the future following quantitative survival estimates, although Mori et al. [57] could not detect a similar effect. None of the included studies discovered a significant impact of explicitness on anxiety, distress, confusion, confidence, insecurity or feeling overwhelmed [54, 57, 60, 64]. Advanced cancer patients rated the provision of statistics about life expectancy evenly hopeful, neutral and not hopeful [68]. In terms of *relational outcomes*, communication between patients, family and professionals was significantly better for patients receiving more specific prognostic information [60]. Furthermore, two experimental studies demonstrated more satisfaction with communication of quantitative instead of qualitative survival estimates [54, 57]. Another paper reported no significant difference in patients’ satisfaction with requested qualitative or supplemental quantitative information [64]. Lastly, studying *care preferences*, explicitness did not affect patients’ willingness to discuss advance care planning with their oncologist [57].

#### Framing of prognostic information

Two experimental studies assessed the effect of framing prognostic information on patient outcomes by incorporating pessimistic, neutral or optimistic statements [69] or using the worst, typical and best case scenario to explain life expectancy [70].

Concerning *information-related outcomes*, one study showed that patients were generally more optimistic about their prognosis than their physicians but were more likely to agree with their oncologist’s estimated chance of cure when physicians made at least one statement of pessimism about the disease outcome [69]. Statements of optimism and uncertainty did not influence physician-patient concordance about the estimated chance of cure [69]. Furthermore, significantly more patients agreed that using the worst, typical and best case scenario to explain life expectancy, versus communicating median survival time, would improve their prognostic understanding, make sense, be helpful, help family and carers and help in making plans for the future [70]. In terms of *psychological outcomes*, significantly more patients agreed that presenting multiple survival scenarios would be reassuring, hopeful and less upsetting and would decrease anxiety, compared with median survival time [70].

#### Affective prognostic communication

Three experimental studies examined the impact of affective prognostic communication on patient outcomes [53, 54, 58]. The effect of affective communication was investigated by comparing respondents’ reactions to video-recorded simulated bad news consultations, including communication about survival and treatment options, with and without physicians’ reassurance of non-abandonment [54] and support [53, 58]. The absence of affective messages was referred to as standard communication [53, 54, 58].

Concerning *information-related outcomes*, one randomised controlled trial discovered better recall during affective versus standard communication [53], while another could not find a similar effect on self-perceived recall ability or actual recall [58]. Regarding *physiological outcomes*, affective communication led to a significantly stronger decrease of physiological arousal compared with standard communication, based on skin conductance levels [53]. Findings on cardiac measures varied, suggesting more sympathetic as well as parasympathetic activity of the autonomic nervous system during standard versus affective communication [58]. Combining *information-related* and *physiological outcomes*, both trials investigated whether the association of physiological arousal with recall differed between the affective and standard communication groups. Based on decreased skin conductance levels [53] and increased parasympathetic activity [58], reduced physiological arousal only led to improved recall during affective communication. However, based on increased heart rate variability, reduced physiological arousal only led to improved recall during standard instead of affective communication [58]. In terms of *psychological outcomes*, anxiety and uncertainty scores were significantly lower following physicians’ reassurance of non-abandonment, while self-efficacy was significantly higher compared with standard communication [54]. Lastly, *relational outcomes* reflected participants’ perceptions of the oncologist’s behaviour in the simulated consultation. Affective communication, as compared to standard communication, led to significantly higher rates of satisfaction, perceived physician’s support, non-abandonment, reassurance and empathy, but not engagement with the patient [53, 54, 58].

#### General communication behaviours

Hagerty and colleagues [68] investigated wide-ranging communication behaviours of physicians, which might influence hope during prognostic discussions. Physicians offering the most up-to-date treatment, appearing to know everything about a patient’s cancer, using humour occasionally, telling pain will be controlled and communicating all treatment options were rated as most hope giving by advanced cancer patients. Those communication behaviours were labelled as expert, positive and collaborative approaches. In contrast, physicians appearing to be nervous, giving prognosis to family first, using euphemisms, avoiding talking about cancer, only discussing treatment and giving good news before bad news were not perceived as hope conveying in this experimental study. Those behaviours were labelled avoidant. Similar to providing survival statistics, physicians’ expression of uncertainty about the disease course was rated evenly hopeful, neutral and not hopeful by metastatic cancer patients [68].

### Moderating factors

Factors moderating the effect of disclosure strategies on patient outcomes were investigated by three studies. Considering *explicit prognostic communication*, patients with a strong monitoring coping style (i.e. often seeking detailed information) were significantly more anxious and uncertain and less self-efficacious and satisfied after explicitness than patients with lower monitoring scores [54]. A blunting coping style (i.e. often avoiding information) did not alter the effect of explicit survival information on patient outcomes [54]. With respect to *framing prognostic information*, higher educated patients were significantly more likely to agree that a typical case scenario, as well as a best case scenario, should be included when communicating life expectancy. Higher education was also associated with agreeing that the best case scenario conveyed hope, and that explaining three scenarios was helpful. No associations were found between education and agreeing that all scenarios were easy to understand [70]. According to the same study, females were significantly more likely to agree that all survival scenarios would help in making plans for the future, and that explaining a worst case scenario was upsetting. Being diagnosed with cancer more than 1 year ago and age younger than 70 were associated with the latter opinion as well. Furthermore, breast cancer patients were significantly less likely to agree that communicating a typical, best and worst case scenario was reassuring, as compared to patients with different primary tumour sites. Instead they were more likely to find this strategy upsetting [70]. Finally, concerning *general communication behaviours*, more anxious patients rated expert, positive, collaborative and empathic approaches to communicate prognosis (e.g. appearing to know everything, working as a team, expressing feelings) as significantly more hope conveying than less anxious patients [68]. The same result was found for older versus younger patients [68].

## Discussion

### Main findings

#### Prognostic disclosure

Integrating the results of all included studies examining prognostic disclosure, and considering methodological quality, evidence shows an association with improved prognostic awareness [22, 59–62]. This suggests that the previously mentioned high rate of prognostic unawareness [13–15] could reasonably be addressed by physicians through prognostic discussions. Moreover, Yun and colleagues [28] illustrate that it might be important for physicians to inform patients of their terminal status, as patients who became aware by their worsening condition or by chance reported lower quality of life.

Addressing oncologists’ worries about harming patients through disclosure [6, 38, 39], the available evidence does not seem to confirm or deny this concern indisputably. Based on two experiments of satisfactory quality, disclosing incurability likely increases immediate physiological arousal [53, 58], indicating that the confrontation with prognostic information affects patients emotionally in that very moment. Literature examining the association of disclosure with longer-term psychological outcomes is limited and of varying quality. Furthermore, these findings are mixed, for which explanations are not found. Taken with caution, however, most point to either no or positive associations between prognostic disclosure and psychological well-being [22, 60, 63–65], which might be reassuring for physicians.

The reported discrepancy between physiological indices of emotional stress and psychological self-report measures could reflect the difference between immediate and delayed responses. Still, it should be taken into consideration that existing measures may not be sensitive enough to capture the complex emotional consequences of prognostic disclosure. Moreover, the effect of prognostic communication is often examined at group level, possibly disregarding subgroups with different reactions.

Accounting for physicians’ worries about the physician-patient relationship [6, 38, 39], two papers show no or positive associations with communicating life expectancy [22, 66]. Additionally, three studies hint to a potential positive association between disclosure and physician-patient communication in general [60] or the discussion of care preferences specifically [56, 61]. Hence, these articles of satisfactory quality suggest that prognostic communication could strengthen the relation and stimulate patients’ role in decision-making [61]. Sufficient evidence for an association between disclosure and the documentation of care preferences, actual care provided or physical outcomes could not be established [56, 60, 61, 67]. Remarkably, even though a key argument for prognostic disclosure is informed decision-making, very few papers investigated the association between prognostic communication and treatment decision-making.

#### Disclosure strategies

The limited number of studies, variety of examined strategies and hypothetical nature of the included experiments complicate drawing conclusions about the effect of different disclosure strategies. Hence, further research is needed to formulate strong recommendations. Based on the existing literature, we tentatively identify four approaches to prognostic communication.

The first strategy encompasses physicians’ provision of more explicit, rather than imprecise, prognostic information. Three studies of satisfactory (and one with lower) quality suggest either no or a beneficial effect of explicitness on patients’ psychological well-being and satisfaction with the consultation [54, 57, 60, 64]. One indicates improved recognition of disease condition [60]. According to the broader literature, being transparent about the difficulty of formulating individual survival estimates is considered helpful when communicating prognosis [44, 54]. Nonetheless, individual differences should be acknowledged, as another paper shows equally large proportions of respondents rating the provision of statistics and communication of uncertainty hopeful, neutral and not hopeful [68].

Second, physicians’ tendency to stress the best case scenario, possibly inducing patients’ overestimation of life expectancy [34, 38, 40, 41], could be complemented with a typical and worst case scenario. Both multiple survival scenarios and pessimistic statements improve patients’ prognostic awareness, according to two studies of satisfactory quality [69, 70]. This realistic strategy might tackle oncologists’ fear of leading patients to focus on a single number [34, 39], help patients to hope for the best, but prepare for the worst [34], and may prevent troublesome consequences of prognostic unawareness [16, 17, 22, 29–33].

A third disclosure strategy involves physicians’ reassurance about non-abandonment and support while communicating life expectancy, investigated by three experiments of satisfactory quality. Findings hint to a beneficial effect of affective communication on patients’ physiological arousal, psychological well-being, satisfaction with the consultation and most measures of perceived physician’s support [53, 54, 58]. The effect of affective communication on recall of information, as well as the joint relation with physiological arousal, remains inconclusive [53, 58]. Nonetheless, this approach should satisfy patients’ need to be looked after and allow for a sense of hope [54].

Fourth, hope might be fostered by physicians through expert, positive and collaborative behaviour during prognostic communication [68]. Incorporating wider research, oncologists are advised to address other sources of hope as well, rather than just medical information, like faith, inner peace, dignity, meaningful life events, relationships or humour [44, 54].

The combination of explicit and affective communication, multiple survival scenarios and expert, positive, collaborative behaviour might be most promising, although more scientific support is needed.

#### Moderating factors

Substantial evidence to identify patient and context characteristics that determine individual reactions to prognostic communication is limited. One study demonstrates that patients who tend to seek detailed information are more anxious and uncertain, and less self-efficacious and satisfied following explicitness [54]. An explanation to this counterintuitive finding might be high monitors’ generally lower satisfaction with information compared with patients showing a low monitoring coping style [54, 71, 72]. Another study suggests that higher educated patients are more likely to find explaining three survival scenarios helpful, and that breast cancer patients are more likely to find this strategy upsetting [70]. An obvious explanation for these findings was not provided, and the authors recommend communicating multiple scenarios to all advanced cancer patients preferring disclosure nevertheless [70]. Lastly, one paper indicates that more anxious and older patients particularly feel hopeful after prognostic communication displaying expertise, positivity, collaboration and empathy [68]. The latter is in line with earlier research, confirming that sensitive communication with emotional support is especially important for older cancer patients [73].

### Limitations

This paper presents a novel attempt to offer comprehensive, evidence-based guidance for physicians’ clinical practice by examining the independent effect of prognostic communication on advanced cancer patients. Despite the suggested disclosure strategies, some patients might prefer and fare better with prognostic ignorance [11, 74, 75]. Reasonably, some experts in the field make a case for prognostic silence, emphasising its protective function [76•, 77]. Exploring a patient’s personal information preferences beforehand, indeed, is essential in prognostic conversations [44, 54]. Caregivers’ information needs should be addressed additionally, as potential dissimilarities could influence prognostic discussions [44].

The reviewed studies have some limitations, which should be taken into account when interpreting the results. Firstly, prognostic disclosure is often assessed with self-report or medical record registration instead of direct observations, which might not reflect the actual extent of prognostic discussions [22, 59]. Secondly, the used study designs entail restrictions. Non-experimental study designs cannot imply causation [63, 66], whereas the controlled context of experiments diminishes the complexity of clinical interactions [54, 57]. Furthermore, respondents acting as analogue patients in experimental studies may react differently than actual patients [53, 54]. Still, since manipulation of prognostic communication in real-life settings is ethically unfeasible, we have to rely on the combination of these study types.

The current paper has shortcomings too. The authors solely searched literature indexed by PubMed and PsycInfo, possibly excluding relevant articles in other databases. Furthermore, attempts to facilitate prognostic communication by means of interventions are not discussed. Studies on advance care planning, early palliative care, decision aids, question prompt lists or communication skills training may provide supplemental information on the effect of prognostic messages.

### Future directions

Considering the limits of literature to-date, future research should expand knowledge about the influence of different prognostic communication formats on patients to convey more pronounced advice, with a focus on psychological well-being over time and treatment decision-making. Studies should attempt to overcome the one-size-fits-all approach, by exploring patients’ individual information preferences and differences in reactions to prognostic communication. Knowledge of moderators, as well as enhanced prognostic prediction models, could assist oncologists in tailoring their messages. Finally, future research should optimise the development and implementation of communication interventions to put guidelines into practice and ultimately improve prognostic communication.

## Conclusions

Altogether, this systematic review synthesised today’s literature on the effect of prognostic communication on patient outcomes in palliative cancer care and moderators of this relation. Addressing the research aims, we conclude cautiously that, if preferred by patients, oncologists can disclose prognosis using sensible strategies. Displaying expertise, positivity and collaboration, while offering explicit prognostic information with multiple survival scenarios and reassurance of support, likely offers an inclusive approach physicians can rely on.

## Opinion statement

Making more deliberate communicative choices starts with physicians’ awareness of their propensity to deliver ambiguous messages. Based on current knowledge and ethical principles, we recommend oncologists to communicate prognosis to patients who wish to know, using a balanced approach. Allowing for preparation as well as hope, we advise mentioning the worst, typical and best case scenario of survival, instead of one number or the optimistic scenario only. Worries about harming patients by truth-telling should not prevent physicians from at least offering prognostic information, as these worries might also originate from personal fears or discomfort. We stimulate physicians to acknowledge patients’ emotions, which often reflect a normal reaction to life-changing messages, and may not necessarily persist. Additionally, we encourage physicians to support patients by reassuring non-abandonment and fostering different types of hope. Oncologists can incorporate the uncertainty of individual estimates by stressing the possible deviation from group-based survival information. Finally, we emphasise the need to individualise care. It is essential to be aware of patients’ frame of reference and to recognise potential differences with one’s own social, cultural and religious context. Discussing prognosis is considered an ongoing process. Therefore, individual information needs have to be explored timely, thoroughly and repeatedly. Subsequently tailoring to patients’ preferences would be most advantageous.

## Appendix 1. Search strategies

### Search strategy PubMed

(Prognosis[Mesh] OR prognos*[tiab] OR prognos*[other term] OR “life expectancy”[Mesh] OR “life expectancy”[tiab] OR mortality[Mesh] OR “disease course”[tiab] OR “mortality risk”[tiab] OR death[Mesh] OR survival[Mesh] OR survival[tiab] OR “bad news”[tiab] OR “truth disclosure”[Mesh] OR “truth disclosure”[tiab]) AND (Communication[Mesh] OR communicat*[tiab] OR communicat*[other term] OR conversat*[tiab] OR messag*[tiab] OR “knowledge transfer”[tiab] OR informing[tiab] OR information[ti] OR “patient education as topic”[Mesh] OR “patient education”[tiab] OR “nonverbal communication”[Mesh] OR “health communication”[Mesh] OR “psychotherapeutic processes”[Mesh] OR discussi*[tiab] OR dialog*[tiab] OR “truth disclosure”[tiab] OR consult*[tiab] OR “professional-patient relations”[Mesh] OR “physician patient relations”[Mesh] OR ((((((patient[tiab] OR client[tiab]))) AND ((physician[tiab] OR specialist[tiab] OR oncologist[tiab] OR professional[tiab] OR doctor[tiab] OR clinician[tiab] OR provider[tiab]))) AND ((interaction*[tiab] OR relation*[tiab] OR communication*[tiab] OR discussion*[tiab])))) OR (((((patient[Other Term] OR client[Other Term]))) AND ((physician[Other Term] OR specialist[Other Term] OR oncologist[Other Term] OR professional[Other Term] OR doctor[Other Term] OR clinician[Other Term] OR provider[Other Term]))) AND ((interaction*[Other Term] OR relation*[Other Term] OR communication*[Other Term] OR discussion*[Other Term])))) AND ((Neoplasms[Mesh] OR neoplasm*[tiab] OR “Medical Oncology”[Mesh] OR oncolog*[tiab] OR oncolog*[Other Term] OR cancer*[tiab] OR carcino*[tiab] OR tumor*[tiab] OR tumour*[tiab] OR sarcoma*[tiab] OR malignan*[tiab] AND “terminal care”[Mesh] OR terminal*[tiab] OR terminal*[Other Term] OR “palliative care”[Mesh] OR palliati*[tiab] OR palliati*[Other Term] OR Neoplasm metastasis[Mesh] OR metasta*[tiab] OR “disease progression”[tiab] OR “terminally ill”[Mesh] OR “advanced cancer”[tiab] OR “advanced cancer”[Other Term] OR “end of life”[tiab] OR “end-of-life”[tiab] OR “supportive care”[tiab] OR incurable[tiab] OR non-curable[tiab])) AND ((Humans[Mesh] AND English[lang] AND (aged, 80 and over[MeSH] OR aged[MeSH] OR middle age[MeSH] OR (middle age[MeSH] OR aged[MeSH]) OR adult[MeSH:noexp] OR adult[MeSH] OR young adult[MeSH])))) AND (Humans[Mesh] AND English[lang] AND (aged, 80 and over[MeSH] OR aged[MeSH] OR middle age[MeSH] OR (middle age[MeSH] OR aged[MeSH]) OR adult[MeSH:noexp] OR adult[MeSH] OR young adult[MeSH]))) Filters: Humans; English; 80 and over: 80+ years; Aged: 65+ years; Middle Aged: 45-64 years; Middle Aged + Aged: 45+ years; Adult: 19-44 years; Adult: 19+ years; Young Adult: 19-24 years

### Search strategy PsycInfo

(((prognosis/ OR prognos*.ab,ti,id. OR “life expectancy”/ OR “life expectancy”.ab,ti. OR “mortality risk”/ OR “mortality risk”.ab,ti. OR “disease course”/ OR prediction/ OR severity/ OR “death and dying”/ OR survival.ab,ti. OR “bad news”.ab,ti. OR “truth disclosure”.ab,ti.) AND (communication/ OR communicat*.ab,ti,id. OR conversation/ OR conversat*.ab,ti. OR messages/ OR messag*.ab,ti. OR “knowledge transfer”/ OR “knowledge transfer”.ab,ti. OR information/ OR informing.ab,ti. OR information.ti. OR “client education”/ OR “patient education”.ab,ti. OR “interpersonal communication”/ OR “verbal communication”/ OR “nonverbal communication”/ OR “oral communication”/ OR “communication skills”/ OR “therapeutic processes”/ OR discussi*.ab,ti. OR dialog*.ab,ti. OR “truth disclosure”.ab,ti. OR consult*.ab,ti. OR ((patient OR client) AND (physician OR specialist OR oncologist OR professional OR doctor OR clinician OR provider) AND (communication* OR discussion* OR interaction* OR relation*)).id,ti,ab.) AND (((exp neoplasms/ NOT “benign neoplasms”/) OR neoplasm*.ab,ti. OR oncology/ OR oncolog*.ab,ti,id. OR cancer*.ab,ti. OR carcino*.ab,ti. OR tumor*.ab,ti. OR tumour*.ab,ti. OR sarcoma*.ab,ti. OR malignan*.ab,ti.) AND (“terminal cancer”/ OR terminal*.ab,ti,id. OR “palliative care”/ OR palliati*.ab,ti,id. OR metastasis/ OR metasta*.ab,ti. OR “disease progression”/ OR “disease progression”.ab,ti. OR “terminally ill patients”/ OR “advanced cancer”.ab,ti,id. OR “end of life”.ab,ti. OR “end-of-life”.ab,ti. OR “supportive care”.ab,ti. OR incurable.ab,ti. OR non-curable.ab,ti.))) AND (adulthood 18 yrs older OR aged 65 yrs older OR middle age 40 64 yrs OR thirties 30 39 yrs OR very old 85 yrs older OR young adulthood 18 29 yrs).ag. AND (dutch OR english).lg.)

## Appendix 2. Quality assessment tools

### Checklist for quality assessment of non-experimental studies

This scale has been adjusted from the adapted Newcastle-Ottawa Scale^1^, to perform a study-specific quality assessment of non-experimental studies for the systematic review of Van der Velden et al. (The Effect of Prognostic Communication on Patient Outcomes in Palliative Cancer Care: a Systematic Review, 2020). A maximum score of 16 points can be attained.

Studies assessed using this scale are as follows: Aoki et al. (1997), Bradley et al. (2001), Cripe et al. (2012), Enzinger et al. (2015), Epstein et al. (2016), Fenton et al. (2018), Fletcher et al. (2013), Nakajima et al. (2012), Robinson et al. (2008), Rumpold et al. (2015), Shin et al. (2016) and Wagner et al. (2010).

*Selection*

1. Representativeness of the sample (0–2 points)^1^

i. Truly representative of the average in the target population (i.e. all subjects or random sampling)**
ii. Somewhat representative of the average in the target population (i.e. non-random sampling)*
iii. Selected group of users (i.e. snowballing or convenience sampling)
iv. No description of the sampling strategy
  2. Sample size (0–2 points)^1^
i. Justified (power analysis) and satisfactory**
ii. Not justified but likely satisfactory (*N* ≥ 128, sample size required to test for a medium-sized mean difference between two independent groups, e.g. disclosure or non-disclosure)*
iii. Justified but not satisfactory, or not justified and likely not satisfactory (*N* < 128)
  3. Non-responders (0–2 points)^1^
i. Response rate is satisfactory (i.e. > 50%) and comparability^2^ of responders’ and non-responders’ characteristics is established**
ii. Response rate is not satisfactory (i.e. < 50%), but comparability of responders’ and non-responders’ characteristics is established*
iii. Response rate is satisfactory (i.e. > 50%), but comparability of responders’ and non-responders’ characteristics is not established
iv. Response rate is not satisfactory (i.e. < 50%) and comparability of responders’ and non-responders’ characteristics is not established, or response rate is not reported

*Comparability*

4. Confounding factors (0–2 points)^1^

i. The study controls for potential confounder(s)**
ii. No control for confounders

*Exposure*

5. Definition of the predictor (prognostic communication) (0–2 points)^3^

i. Definition of the predictor is clearly described**
ii. Unclear or no description of the definition of the predictor
  6. Assessment of the predictor (prognostic communication) (0–2 points)^3^
i. Observation of real consultations (coding of audio-recorded visits)**
ii. Tailoring of real consultations or medical record registration*
iii. Self-report by doctors or patients of real consultations
iv. No description of assessment of the predictor

*Outcome*

7. Validation of the measurement tools for patient outcomes (0–2 points)^1^

i. Validated measurement tools (self-report or structured interview) or medical record registration of DNRs**
ii. Non-validated measurement tools included (self-report, interview question or medical record registration of conversations), but the tools are all available or described*
iii. No description of measurement tools
  8. Statistical test (0–2 points)^1^
i. The statistical test used to analyse the data is clearly described and is appropriate (including multilevel analyses if possible and deemed necessary, and corrections for multiple testing when > 20 relations were tested), and all significant and non-significant results are reported including the measurement of association and *p* values or confidence intervals**
ii. Two out of three of the above present*
iii. The statistical test is not appropriate, incomplete or not described

*Total score*

^1^Adjusted from the adapted Newcastle-Ottawa Scale

^2^Comparability is considered established when responders have been compared with non-responders or with the total population, and no significant differences were found

^3^Self-constructed item specified to suit the research questions of the systematic review of Van der Velden et al. (The Effect of Prognostic Communication on Patient Outcomes in Palliative Cancer Care: a Systematic Review, 2020)

### Checklist for quality assessment of experimental studies

This is a self-constructed checklist to perform a study-specific quality assessment of experimental studies for the systematic review of Van der Velden et al. (The Effect of Prognostic Communication on Patient Outcomes in Palliative Cancer Care: a Systematic Review, 2020), consisting of two parts. Experimental studies using a within-subjects design are assessed with part A. Experimental studies using a between-subjects design are assessed with parts A and B. Items of the Cochrane Consumer and Communication Review Group criteria, the Cochrane Collaboration’s tool for assessing risk of bias and the adapted Newcastle-Ottawa Scale were adjusted and used for the current checklist. A maximum score of 8 points can be attained using part A only. A maximum score of 13 points can be attained using parts A and B.

Studies assessed using this scale are as follows: Danzi et al. (2018), Hagerty et al. (2005), Kiely et al. (2013), Mori et al. (2019), Sep et al. (2014) and Van Vliet et al. (2013).

*Part A*

1. Was the definition of the predictor (prognostic communication) clearly described?^1^
2. Was the manipulation of the predictor (prognostic communication) clearly described?^1^
3. Was the data analyst blinded (i.e. was the code only broken after conclusions were drawn)?^3, 6^
4. Were no outcome data missing, or were missing outcome data unlikely to be related to true outcome, balanced across conditions with similar reasons, assumed not to have a clinically relevant impact on the intervention effect or imputed with appropriate methods?^3^
5. Were validated tools (i.e. well-known questionnaires, reliable coding schemes or standardised instruments for physiological measurements) used to assess all outcomes that were related to prognostic communication or were non-validated tools at least all available or described?^2^
6. Was the sample size justified and satisfactory, or not justified but presumably satisfactory (*N* ≥ 128, sample size required to test for a medium-sized mean difference between two independent groups, e.g. disclosure or non-disclosure)?^5^
7. Was the response rate satisfactory (i.e. > 50%) and was comparability of responders’ and non-responders’ characteristics established?^5, 7^
8. Was the statistical test used to analyse the data clearly described and appropriate (including multilevel analyses if possible and deemed necessary, and corrections for multiple testing when > 20 relations were tested), and were all significant and non-significant results reported including the measurement of association and *p* values or confidence intervals?^5^

*Part B*

9. Was the method of randomisation adequate (i.e. truly random)?^2, 6^
10. Was allocation concealed (i.e. could allocation to conditions have been influenced)?^2, 6^
11. Was the (analogue) patient blinded (i.e. did the patient know to what condition s(he) was allocated)?^2^
12. Were groups compared at baseline on at least one potential determinant of the outcomes (i.e. socio-demographics, medical characteristics and primary outcomes) and were the analyses properly controlled for differences?^2^
13. With the exception of the trial intervention, were the experimental and control condition equivalent (i.e. was a placebo added to the control condition)?^4^

*Total score*

^1^Self-constructed item specified to suit the research questions of the systematic review of Van der Velden et al. (The Effect of Prognostic Communication on Patient Outcomes in Palliative Cancer Care: a Systematic Review, 2020)

^2^Among the criteria formulated by the Cochrane Consumer and Communication Review Group

^3^Adjusted from the Cochrane Collaboration’s tool for assessing risk of bias

^4^Adopted from Henselmans et al. [50]

^5^Adjusted from the adapted Newcastle-Ottawa Scale

^6^Considered fulfilled when called random, concealed or blinded by authors or when the text explicitly refers to such methods (such as mention of ‘sealed opaque envelopes’, a cover story for patients in the control group or separate consent forms)

^7^Comparability is considered established when responders have been compared with non-responders or with the total population, and no significant differences were found

## Appendix 3

**Table 2:** Quality assessment of non-experimental studies

Authors (year)	Selection			Comparability	Exposure		Outcome		Total and % of maximum score
	(1) Representativeness of the sample	(2) Power and sample size	(3) Non-response	(4) Confounders	(5) Definition of prognosis	(6) Assessment of predictor	(7) Validated tools	(8) Statistics	
Aoki et al. (1997)	–	–	–	–	–	*	**	*	4/16 = 25.0%
Bradley et al. (2001)	*	*	**	**	**	**	*	**	13/16 = 81.3%
Cripe et al. (2012)	–	–	–	**	**	–	**	*	7/16 = 43.8%
Enzinger et al. (2015)	*	*	**	**	**	–	*	*	10/16 = 62.5%
Epstein et al. (2016)	*	**	–	**	**	–	*	*	9/16 = 56.3%
Fenton et al. (2018)	*	*	–	**	**	**	**	**	12/16 = 75.0%
Fletcher et al. (2013)	*	–	–	**	**	–	*	*	7/16 = 43.8%
Nakajima et al. (2012)	*	–	**	**	**	*	**	**	12/16 = 75.0%
Robinson et al. (2008)	*	*	–	**	**	**	*	**	11/16 = 68.8%
Rumpold et al. (2015)	*	–	–	–	**	*	*	*	6/16 = 37.5%
Shin et al. (2016)	*	*	–	**	**	–	**	**	10/16 = 62.5%
Wagner et al. (2010)	*	*	–	**	**	–	*	*	8/16 = 50.0%

A maximum of 2 points can be attained per item

## Appendix 4

**Table 3:** Quality assessment of experimental studies

Authors (year)	Part A								Part B					Total and % of maximum score
	(1) Definition of prognosis	(2) Manipulation of predictor	(3) Blinded data analyst	(4) Missing outcome data	(5) Validated tools	(6) Power and sample size	(7) Non-response	(8) Statistics	(9) Randomisation	(10) Allocation	(11) Blinded patient	(12) Group comparison	(13) Equivalent conditions	
Non-controlled experimental studies (within-subjects design)														
Hagerty et al. (2005)	–	*	–	*	*	–	*	–	NA	NA	NA	NA	NA	4/8 = 50.0%
Kiely et al. (2013)	*	*	–	*	*	*	–	–	NA	NA	NA	NA	NA	5/8 = 62.5%
Mori et al. (2019)	*	*	–	*	*	*	–	*	NA	NA	NA	NA	NA	6/8 = 75.0%
Van Vliet et al. (2013)	*	*	–	*	*	*	–	*	NA	NA	NA	NA	NA	6/8 = 75.0%
Controlled experimental studies (between-subjects design)														
Danzi et al. (2018)	–	*	–	*	–	–	–	*	*	*	*	*	*	8/13 = 61.5%
Sep et al. (2014)	*	*	–	*	–	–	–	*	*	*	*	*	*	9/13 = 69.2%

A maximum of 1 point can be attained per item

## References

[1] Gerber DE (2008). Targeted therapies: a new generation of cancer treatments. Am Fam Physician..

[2] Kanavos P (2006). The rising burden of cancer in the developing world. Ann Oncol.

[3] Bray F, Ferlay J, Soerjomataram I, Siegel RL, Torre LA, Jemal A (2018). Global cancer statistics 2018: GLOBOCAN estimates of incidence and mortality worldwide for 36 cancers in 185 countries. CA Cancer J Clin.

[4] LeBlanc TW, Temel JS, Helft PR (2018). “How much time do I have?”: communicating prognosis in the era of exceptional responders. Am Soc Clin Oncol Educ Book.

[5] Owen R, Jeffrey D (2008). Communication: common challenging scenarios in cancer care. Eur J Cancer.

[6] Gordon EJ, Daugherty CK (2003). ‘Hitting you over the head’: oncologists’ disclosure of prognosis to advanced cancer patients. Bioethics..

[7] Gramling R, Fiscella K, Xing G, Hoerger M, Duberstein P, Plumb S, Mohile S, Fenton JJ, Tancredi DJ, Kravitz RL, Epstein RM (2016). Determinants of patient-oncologist prognostic discordance in advanced cancer. JAMA Oncol.

[8] Rodriguez KL, Gambino FJ, Butow PN, Hagerty RG, Arnold RM (2008). ‘It’s going to shorten your life’: framing of oncologist-patient communication about prognosis. Psychooncology..

[9] Quirt CF, Mackillop WJ, Ginsburg AD, Sheldon L, Brundage M, Dixon P, Ginsburg L (1997). Do doctors know when their patients don’t? A survey of doctor-patient communication in lung cancer. Lung Cancer.

[10] Jackson VA, Jacobsen J, Greer JA, Pirl WF, Temel JS, Back AL (2013). The cultivation of prognostic awareness through the provision of early palliative care in the ambulatory setting: a communication guide. J Palliat Med.

[11] Hagerty RG, Butow PN, Ellis PA, Lobb EA, Pendlebury S, Leighl N (2004). Cancer patient preferences for communication of prognosis in the metastatic setting. J Clin Oncol.

[12] Harding R, Simms V, Calanzani N, Higginson IJ, Hall S, Gysels M, Meñaca A, Bausewein C, Deliens L, Ferreira P, Toscani F, Daveson BA, Ceulemans L, Gomes B, PRISMA (2013). If you had less than a year to live, would you want to know? A seven-country European population survey of public preferences for disclosure of poor prognosis. Psycho-Oncology..

[13] Liu PH, Landrum MB, Weeks JC, Huskamp HA, Kahn KL, He YL (2014). Physicians’ propensity to discuss prognosis is associated with patients’ awareness of prognosis for metastatic cancers. J Palliat Med.

[14] Mack JW, Walling A, Dy S, Antonio ALM, Adams J, Keating NL, Tisnado D (2015). Patient beliefs that chemotherapy may be curative and care received at the end of life among patients with metastatic lung and colorectal cancer. Cancer..

[15] Trevino KM, Zhang BH, Shen MJ, Prigerson HG (2016). Accuracy of advanced cancer patients’ life expectancy estimates: the role of race and source of life expectancy information. Cancer..

[16] Temel JS, Greer JA, Admane S, Gallagher ER, Jackson VA, Lynch TJ (2011). Longitudinal perceptions of prognosis and goals of therapy in patients with metastatic non-small-cell lung cancer: results of a randomized study of early palliative care. J Clin Oncol.

[17] Weeks JC, Cook EF, O’Day SJ, Petersen LM, Wenger N, Reding D (1998). Relationship between cancer patients’ predictions of prognosis and their treatment preferences. JAMA.

[18] Weeks JC, Catalano PJ, Cronin A, Finkelman MD, Mack JW, Keating NL, Schrag D (2012). Patients’ expectations about effects of chemotherapy for advanced cancer. N Engl J Med.

[19] Fried TR, Bradley EH, O’Leary J (2006). Changes in prognostic awareness among seriously ill older persons and their caregivers. J Palliat Med.

[20] Chen CH, Wen FH, Hou MM, Hsieh CH, Chou WC, Chen JS (2017). Transitions in prognostic awareness among terminally ill cancer patients in their last 6 months of life examined by multi-state Markov modeling. Oncologist.

[21] Baek SK, S-y K, Heo DS, Yun YH, Lee MK (2012). Effect of advanced cancer patients’ awareness of disease status on treatment decisional conflicts and satisfaction during palliative chemotherapy: a Korean prospective cohort study. Support Care Cancer.

[22] Enzinger AC, Zhang B, Schrag D, Prigerson HG (2015). Outcomes of prognostic disclosure: associations with prognostic understanding, distress, and relationship with physician among patients with advanced cancer. J Clin Oncol.

[23] Hui D, Con A, Christie G, Hawley PH (2009). Goals of care and end-of-life decision making for hospitalized patients at a Canadian tertiary care cancer center. J Pain Symptom Manage.

[24] Lee MK, Baek SK, S-y K, Heo DS, Yun YH, Park SR (2013). Awareness of incurable cancer status and health-related quality of life among advanced cancer patients: a prospective cohort study. Palliat Med.

[25] Tang ST, Liu TW, Chow JM, Chiu CF, Hsieh RK, Chen CH, Liu LN, Feng WL (2014). Associations between accurate prognostic understanding and end-of-life care preferences and its correlates among Taiwanese terminally ill cancer patients surveyed in 2011–2012. Psychooncology..

[26] Tang ST, Liu TW, Tsai CM, Wang CH, Chang GC, Liu LN (2008). Patient awareness of prognosis, patient–family caregiver congruence on the preferred place of death, and caregiving burden of families contribute to the quality of life for terminally ill cancer patients in Taiwan. Psychooncology..

[27] Tchen N, Bedard P, Yi Q, Klein M, Cella D, Eremenco S, Tannock IF (2003). Quality of life and understanding of disease status among cancer patients of different ethnic origin. Br J Cancer.

[28] Yun YH, Kwon YC, Lee MK, Lee WJ, Jung KH, Do YR (2010). Experiences and attitudes of patients with terminal cancer and their family caregivers toward the disclosure of terminal illness. J Clin Oncol.

[29] Fried TR, Bradley EH, Towle VR, Allore H (2002). Understanding the treatment preferences of seriously ill patients. N Engl J Med.

[30] Prigerson HG (1992). Socialization to dying—social determinants of death acknowledgment and treatment among terminally ill geriatric-patients. J Health Soc Behav.

[31] Cherlin E, Fried T, Prigerson HG, Schulman-Green D, Johnson-Hurzeler R, Bradley EH (2005). Communication between physicians and family caregivers about care at the end of life: when do discussions occur and what is said?. J Palliat Med.

[32] Friedman BT, Harwood MK, Shields M (2002). Barriers and enablers to hospice referrals: an expert overview. J Palliat Med.

[33] Wright AA, Mack JW, Kritek PA, Balboni TA, Massaro AF, Matulonis UA (2010). Influence of patients’ preferences and treatment site on cancer patients’ end-of-life care. Cancer.

[34] Henselmans I, Smets EMA, Han PKJ, de Haes H, Laarhoven H (2017). How long do I have? Observational study on communication about life expectancy with advanced cancer patients. Patient Educ Couns.

[35] Cartwright C, Onwuteaka-Philipsen BD, Williams G, Faisst K, Mortier F, Nilstun T (2007). Physician discussions with terminally ill patients: a cross-national comparison. Palliat Med.

[36] Gattellari M, Voigt KJ, Butow PN, Tattersall MH (2002). When the treatment goal is not cure: are cancer patients equipped to make informed decisions?. J Clin Oncol..

[37] Koedoot CG, Oort FJ, de Haan RJ, Bakker PJM, de Graeff A, De Haes JCJM (2004). The content and amount of information given by medical oncologists when telling patients patients with advanced cancer what their treatment options are: palliative chemotherapy and watchful-waiting. Eur J Cancer.

[38] Clayton JM, Hancock K, Parker S, Butow PN, Walder S, Carrick S (2008). Sustaining hope when communicating with terminally ill patients and their families: a systematic review. Psychooncology.

[39] Hancock K, Clayton JM, Parker SM, Wal DS, Butow PN, Carrick S (2007). Truth-telling in discussing prognosis in advanced life-limiting illnesses: a systematic review. Palliat Med.

[40] Christakis NA (2001). Death foretold: prophecy and prognosis in medical care.

[41] Loprinzi CL, Johnson ME, Steer G (2003). Doc, how much time do I have?. J Clin Oncol.

[42] The AM, Hak T, Koeter G, van Der WG (2000). Collusion in doctor-patient communication about imminent death: an ethnographic study. BMJ..

[43] Chen CH, Kuo SC, Tang ST (2017). Current status of accurate prognostic awareness in advanced/terminally ill cancer patients: systematic review and meta-regression analysis. Palliat Med.

[44] Clayton JM, Hancock KM, Butow PN, Tattersall MH, Currow DC, Adler J, et al. Clinical practice guidelines for communicating prognosis and end-of-life issues with adults in the advanced stages of a life-limiting illness, and their caregivers. Med J Aust. 2007;186(12 Suppl):S77 S9, S83-108. 10.5694/j.1326-5377.2007.tb01100.x 17727340

[45] Hagerty RG, Butow PN, Ellis PM, Dimitry S, Tattersall MH (2005). Communicating prognosis in cancer care: a systematic review of the literature. Ann Oncol.

[46] Moher D, Liberati A, Tetzlaff J, Altman DG, PRISMA Group (2009). Preferred Reporting Items for Systematic Reviews and Meta-Analyses: the PRISMA statement. PLoS Med.

[47] Wells G, Shea B, O’Connell D, Peterson J, Welch V, Losos M et al. Newcastle-Ottawa quality assessment scale cohort studies. 2014.

[48] Hillen MA, Medendorp NM, Daams JG, Smets EM (2017). Patient-driven second opinions in oncology: a systematic review. Oncologist.

[49] Ryan R, Hill S, Broclain D, Horey D, Oliver S, Prictor M. Cochrane Consumers and Communication Review Group. Study Quality Guide. 2007.

[50] Henselmans I, de Haes HC, Smets EM (2013). Enhancing patient participation in oncology consultations: a best evidence synthesis of patient-targeted interventions. Psychooncology..

[51] Sardell AN, Trierweiler SJ. Disclosing the cancer diagnosis. Procedures that influence patient hopefulness. Cancer. 1993;72(11):3355–3365. 10.1002/1097-0142(19931201)72:11<3355::aid-cncr2820721135>3.0.co;2-d 8242563

[52] Butow P, Dowsett S, Hagerty R, Tattersall M. Communicating prognosis to patients with metastatic disease: what do they really want to know?. Supportive Care in Cancer. 2002;10(2):161–168. 10.1007/s005200100290 11862506

[53] Sep MS, Van Osch M, Van Vliet LM, Smets EM, Bensing JM (2014). The power of clinicians’ affective communication: how reassurance about non-abandonment can reduce patients’ physiological arousal and increase information recall in bad news consultations. An experimental study using analogue patients. Patient Educ Couns.

[54] Van Vliet LM, van der Wall E, Plum NM, Bensing JM (2013). Explicit prognostic information and reassurance about nonabandonment when entering palliative breast cancer care: findings from a scripted video-vignette study. J Clin Oncol.

[55] Van Vliet L, Francke A, Tomson S, Plum N, van der Wall E, Bensing J (2013). When cure is no option: how explicit and hopeful can information be given? A qualitative study in breast cancer. Patient Educ Couns.

[56] Bradley EH, Hallemeier AG, Fried TR, Johnson-Hurzeler R, Cherlin EJ, Kasl SV, Horwitz SM (2001). Documentation of discussions about prognosis with terminally ill patients. Am J Med.

[57] Mori M, Fujimori M, van Vliet LM, Yamaguchi T, Shimizu C, Kinoshita T, et al. Explicit prognostic disclosure to Asian women with breast cancer: a randomized, scripted video-vignette study (J-SUPPORT1601). Cancer. 2019. 10.1002/cncr.32327. 10.1002/cncr.32327 31206639

[58] Danzi OP, Perlini C, Tedeschi F, Nardelli M, Greco A, Scilingo EP, Valenza G, del Piccolo L (2018). Affective communication during bad news consultation. Effect on analogue patients’ heart rate variability and recall. Patient Educ Couns.

[59] Epstein AS, Prigerson HG, O’Reilly EM, Maciejewski PK. Discussions of life expectancy and changes in illness understanding in patients with advanced cancer. J Clin Oncol. 2016. 10.1200/JCO.2015.63.6696. 10.1200/JCO.2015.63.6696 PMC4981977 27217454

[60] Nakajima N, Hata Y, Onishi H, Ishida M (2012). The evaluation of the relationship between the level of disclosure of cancer in terminally ill patients with cancer and the quality of terminal care in these patients and their families using the Support Team Assessment Schedule. Am J Hospice Palliat Med.

[61] Wagner GJ, Riopelle D, Steckart J, Lorenz KA, Rosenfeld KE (2010). Provider communication and patient understanding of life-limiting illness and their relationship to patient communication of treatment preferences. J Pain Symptom Manag.

[62] Fletcher K, Prigerson HG, Paulk E, Temel J, Finlay E, Marr L (2013). Gender differences in the evolution of illness understanding among patients with advanced cancer. J Support Oncol.

[63] Cripe LD, Rawl SM, Schmidt KK, Tong Y, Monahan PO, Rand KL (2012). Discussions of life expectancy moderate relationships between prognosis and anxiety or depression in men with advanced cancer. J Palliat Med.

[64] Rumpold T, Lütgendorf-Caucig C, Jagsch R, Dieckmann K, Watzke H, Pötter R, Kirchheiner K (2015). Information preferences regarding cure rates and prognosis of Austrian patients with advanced lung cancer. Strahlenther Onkol.

[65] Shin JA, El-Jawahri A, Parkes A, Schleicher SM, Knight HP, Temel JS (2016). Quality of life, mood, and prognostic understanding in patients with metastatic breast cancer. J Palliat Med.

[66] Fenton JJ, Duberstein PR, Kravitz RL, Xing G, Tancredi DJ, Fiscella K, Mohile S, Epstein RM (2018). Impact of prognostic discussions on the patient-physician relationship: prospective cohort study. J Clin Oncol.

[67] Aoki Y, Nakagawa K, Hasezawa K, Tago M, Baba N, Toyoda K, Toyoda T, Kozuka T, Kiryu S, Igaki H, Sasaki Y (1997). Significance of informed consent and truth-telling for quality of life in terminal cancer patients. Radiat Med.

[68] Hagerty RG, Butow PN, Ellis PM, Lobb EA, Pendlebury SC, Leighl N (2005). Communicating with realism and hope: incurable cancer patients’ views on the disclosure of prognosis. J Clin Oncol.

[69] Robinson TM, Alexander SC, Hays M, Jeffreys AS, Olsen MK, Rodriguez KL, Pollak KI, Abernethy AP, Arnold R, Tulsky JA (2008). Patient–oncologist communication in advanced cancer: predictors of patient perception of prognosis. Support Care Cancer.

[70] Kiely BE, McCaughan G, Christodoulou S, Beale PJ, Grimison P, Trotman J, Tattersall MH, Stockler MR (2013). Using scenarios to explain life expectancy in advanced cancer: attitudes of people with a cancer experience. Support Care Cancer.

[71] Miller SM, Mangan CE (1983). Interacting effects of information and coping style in adapting to gynecologic stress: should the doctor tell all?. J Pers Soc Psychol.

[72] Steptoe A, Sutcliffe I, Allen B, Coombes C (1991). Satisfaction with communication, medical knowledge, and coping style in patients with metastatic cancer. Soc Sci Med.

[73] Posma ER, van Weert JCM, Jansen J, Bensing JM (2009). Older cancer patients’ information and support needs surrounding treatment: an evaluation through the eyes of patients, relatives and professionals. BMC Nurs.

[74] Barnett MM (2006). Does it hurt to know the worst?—psychological morbidity, information preferences and understanding of prognosis in patients with advanced cancer. Psychooncology..

[75] Innes S, Payne S (2009). Advanced cancer patients’ prognostic information preferences: a review. Palliat Med.

[76] Han PK (2016). The need for uncertainty: a case for prognostic silence. Perspect Biol Med.

[77] Helft PR (2005). Necessary collusion: prognostic communication with advanced cancer patients. J Clin Oncol.

